# Color preference of the spotted wing Drosophila, *Drosophila suzukii*

**DOI:** 10.1038/s41598-019-52425-w

**Published:** 2019-11-05

**Authors:** Catherine M. Little, A. Rebecca Rizzato, Lise Charbonneau, Thomas Chapman, N. Kirk Hillier

**Affiliations:** 1 Department of Biology, Acadia University, B4P2R6 Wolfville, NS Canada; 2 Department of Biology, Memorial University of Newfoundland and Labrador, A1C5S7 St. John’s, NL Canada

**Keywords:** Behavioural ecology, Evolutionary ecology, Colour vision

## Abstract

*Drosophila suzukii* Matsumura (Diptera: Drosophilidae) is a significant invasive pest in soft-skin fruits and berries in Asia, Europe, and North and South America. Many herbivorous insects use multiple cues for host selection, particularly olfactory and visual stimuli. The visual system of closely-related *Drosophila melanogaster* is well-documented, expressing strong sensitivity to short-wavelength colors (ultraviolet to green) and only limited sensitivity to long-wavelength colors (red to infrared). Our results suggest that *D. suzukii* have limited ability to distinguish red consistent with visual sensitivity range within the melanogaster subgroup. We propose that color contrast rather than color appearance may be of greater importance in orientation and attraction. We propose that differences in reflectance between light wavelengths important for color opponency are key to color discrimination to provide color contrast between foreground and background, as occurs between fruit and foliage, during host-finding.

## Introduction

Host-finding by insects often relies on the integration of a combination or sequential reception of olfactory, visual, tactile, and/or gustatory cues to identify suitable hosts^1,2^. Use of multi-modal cues for host-finding is widespread, and hierarchical sensory systems have been identified in numerous species from several insect orders, including Lepidoptera^3–5^, Hymenoptera^6,7^, Coleoptera^8^, and Diptera^9^. Even within a single insect species, separate host-races of *Rhagoletis pomonella* (apple versus hawthorn) can be distinguished by differences in attraction to both olfactory and visual cues^10^.

The spotted wing Drosophila, *Drosophila suzukii* Matsumura (Diptera: Drosophilidae), is thought to be endemic to South-East Asia and is a highly polyphagous invasive pest insect in Asia, North America, South America, and Europe^11–17^. Female *D. suzukii* use their serrated ovipositor to lay eggs in soft-skinned fruits and berries, resulting in millions of dollars in damage to fruit crops^18,19^. Volatile organic compounds associated with ripening fruits and naturally occurring yeasts have been widely acknowledged as key factors in host-finding behaviour for *Drosophila* species, including *D. suzukii*^20–22^. Visual cues are also important to host-finding behaviour^23^. To that end, monitoring traps in use for *D. suzukii* are red or employ a combination of black and red^24–27^. Recent research supports the attractiveness of red and black against a white background^28^. However, monitoring traps used in fruit crops are normally deployed amongst foliage rather than a white background. This may explain why monitoring traps in a combination of clear plastic and yellow have been used with similar efficacy^25,29,30^. Previous research has demonstrated that color contrast between foreground and background can facilitate food search efforts by frugivorous birds, pollinators (florivorous birds and insects), and host search efforts by Tephritid flies^31,32^. Similar mechanisms may play a role in host-finding by *D. suzukii*.

Color vision can be defined as the ability to discriminate among color stimuli based on wavelength composition and independent of intensity (or brightness)^33,34^. Color vision in a closely related species, *Drosophila melanogaster*, has been studied extensively^35–37^. *Drosophila* species are thought to be most sensitive to ultraviolet, blues, and greens^35,36,38–40^. Peak sensitivity in *D. melanogaster* occurs at 420 nm and 495 nm; however, visual sensitivity is relatively stable and consistent from 406 nm to 525 nm^41^. Sensitivity drops rapidly at longer wavelengths, with up to 25 times less sensitivity at 606 nm than at 505 nm^41^. Thus *D. melanogaster* are most sensitive to light of shorter wavelengths (ultraviolet, blue, and green), with only limited sensitivity to light of longer wavelengths (orange, red, and infrared). The color vision system of *Drosophila* spp. is thought to be highly conserved^38^.

The compound eye of *D. melanogaster* contains eight different photoreceptors expressing five spectrally distinct types of opsins^37^. In *D. melanogaster*, inner photoreceptors R7 and R8 are sufficient to distinguish between blue and green and provide limited color discrimination over a wider range^37^. Four types of opsins are expressed on the inner photoreceptors R7 and R8. At the eye margin, both R7 and R8 express opsin Rh3, sensitive to ultraviolet. Elsewhere in the eye, R7 and R8 photoreceptors come in two forms, pale (p) and yellow (y). In pale forms, R7 expresses Rh3 (ultraviolet) and R8 expresses Rh5 (blue). In yellow forms, R7 expresses Rh4 (longer UV wavelengths) and R8 expresses Rh6 (green)^35^. However, broader spectrum color discrimination requires input from outer photoreceptors (photoreceptors R1-R6), expanding visual sensitivity range to between 306 nm and 540 nm. Photoreceptors R1-R6 express the same type of opsin (Rh1), which is broadly tuned to blue and ultraviolet light. These outer photoreceptors are critical for motion detection and vision under low light conditions. Distinction of color by *D. melanogaster* requires stimulation of two or more photoreceptors of different spectral sensitivities; however, all photoreceptors in the *D. melanogaster* eye are selectively tuned to the ultraviolet to green, effectively limiting color vision to the shorter wavelengths. Thus, color vision in *D. melanogaster* occurs via “interommatidial” opponency photoreceptors (i.e., Rh3-Rh4 and Rh5-Rh6 in R7 and R8) and a possible additional opponency dimension from outer photoreceptors (Rh1 in R1-R6 interacting with Rh4 in R7) which serve to enhance color discrimination^37^. Although most long-wavelength light is reflected by the *D. melanogaster* eye, small amounts of red light can enter the eye at an oblique angle to re-sensitize photosensitive pigments enabling increased sensitivity to ultraviolet^34,42^. Even accounting for the shift in spectral sensitivity due to retinal pigments, *D. melanogaster* are not able to distinguish distinct colors above 600 nm^43^. Therefore, it is unsurprising that *D. melanogaster* phototactic behaviour reflects a preference for short wavelength (UV to green) light over long wavelength (red) light by almost 2 orders of magnitude^44^.

Studies of color preference of insect models differ depending on whether and how colors are quantified or described^45,46^. Numerous methods of quantifying color are currently in use, mostly based on human perception of color appearance^47^. Concepts of color brightness, hue, chroma, and saturation are comparative measures of color perception based on human color vision and can be influenced by the viewers assumptions about environmental conditions including assumed illumination of the object viewed^33,34,47,48^. Alternatively, the XYZ color space model is also frequently used to quantify color in animal color perception studies although it is also based on human color perception and requires identical viewing conditions, including illumination and background, to compare differences among colors^47^. Therefore, we have used measures of light wavelength and reflected wavelength, which are objective independent of the species perceiving the color and of viewing conditions. To ensure that color perception by subject *D. suzukii* is consistent, all assays involving LED light colors were conducted in the absence of extraneous light and all choice assays using foam and cardstock colors were completed using identical lighting conditions consistent with standardized natural daylight conditions (https://www.yujiintl.com/high-cri-led-lighting.html).

The goal of this study was to confirm the relative sensitivity and investigate the preference of *D. suzukii* to reflected light from a range of colored targets to optimize monitoring and trapping efficacy. We tested sensitivity of *D. suzukii* to different colors of light, the relative attractiveness of colored light, and of reflected colors alone and in combination. Visual spectral sensitivity is thought to be highly conserved within *Drosophila* species and to a lesser extent, insects in general^38^. Thus, it is probable that visual sensitivity ranges are similar between *D. suzukii* and *D. melanogaster*. We previously demonstrated that *D. suzukii* are highly attuned to changes in foliage colors and are attracted to fruit colors which color contrast against foliage colors^23^. This suggests that color contrast between foreground and background colors, as is found between fruit and foliage, may be a key factor in host-finding behaviour.

## Results

### Sensitivity to color (Electroretinography)

**Figure 1:**
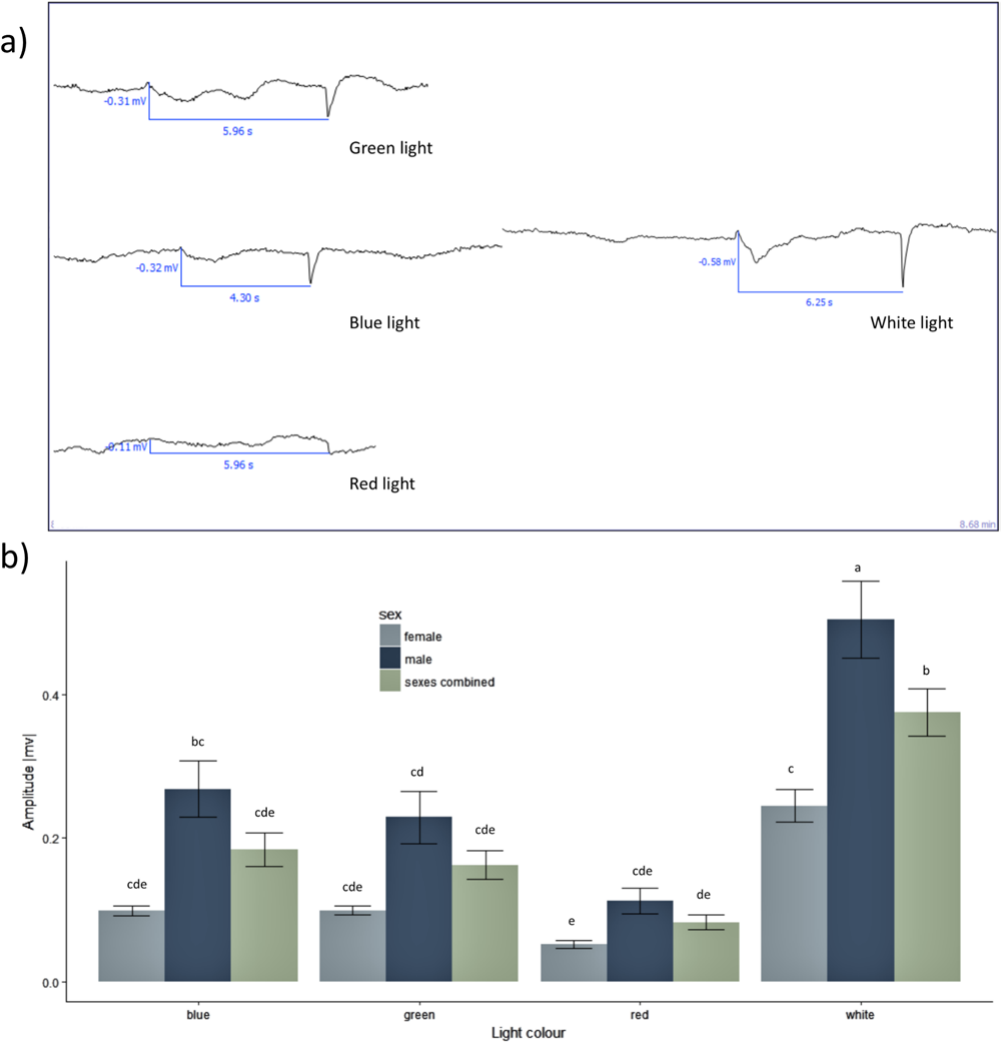
(**a**) Representative electroretinogram responses to white and colored lights. (**b**) Mean (±SEM) electroretinogram results for each light color by *D. suzukii* sex. Different letters denote significant differences light colors.

Significant differences were observed among *D. suzukii* responses to white and colored lights (Fig. 1a,b). Light color, sex of the *D. suzukii*, and the interaction of these factors all contributed significantly to differences in physiological responses (2-way ANOVA; Color: F_3,191_ = 32.64, P < 0.001; Sex_1,191_ = 51.22, P < 0.001; Color:Sex: F_3,191_ = 3.53, P = 0.02) (Fig. 1b). Responses from male *D. suzukii* were consistently stronger than from females (ANOVA, F_1,197_ = 33.96, P < 0.0001). Combining responses of male and female flies show that mean electroretinogram values are higher for blue light than for red light (1-way ANOVA; F_3,195_ = 25.18, P < 0.0001) (Fig. 1b).

### Preference among LED light colors

**Figure 2:**
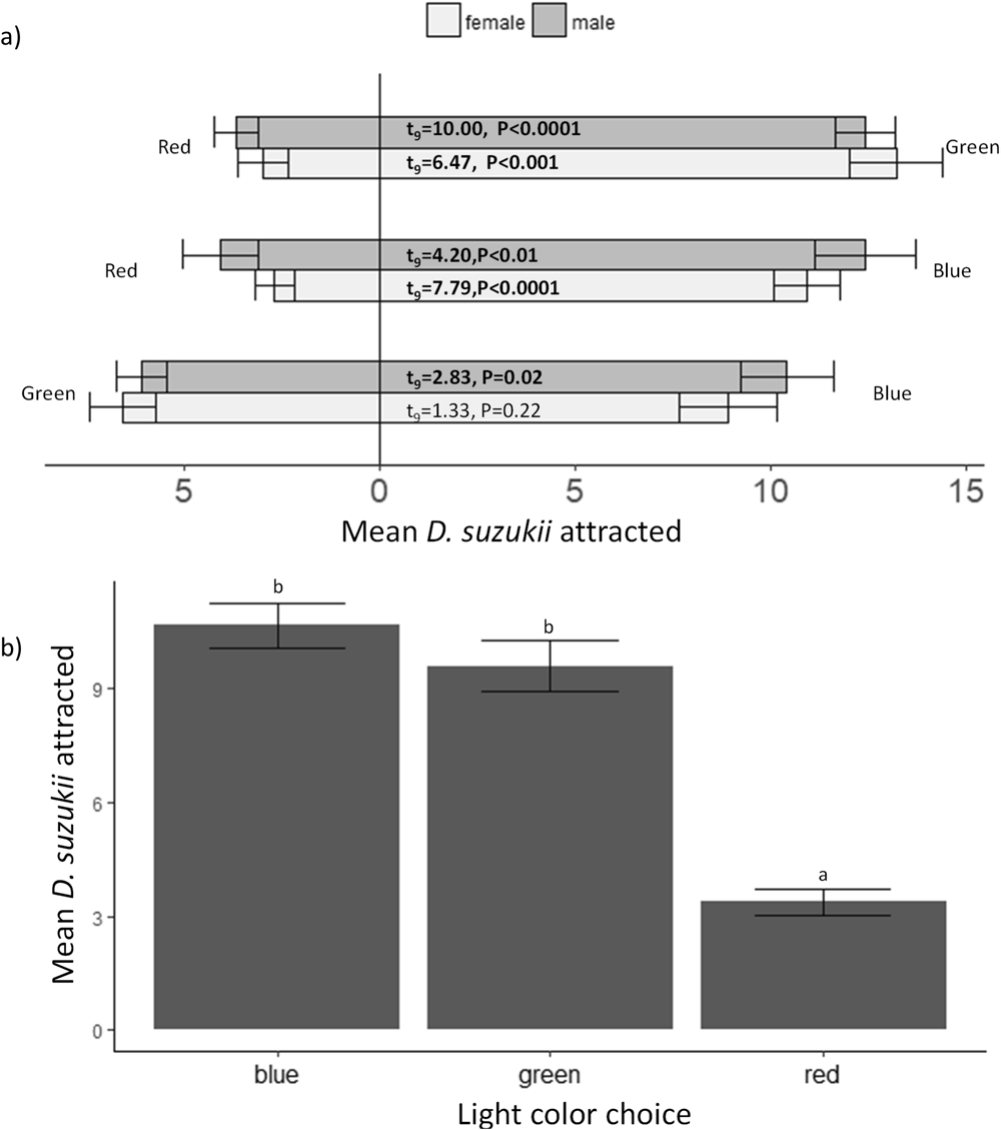
(**a**) Mean count (±SE) of *D. suzukii* attracted to colored lights in 2-choice trials. Results of Paired t-tests are shown within the figure (significant differences are in bold). (**b**) Mean counts of male and female *D. suzukii* attracted to each color in 2-choice assays.

Differences between responses by females and males in 2-choice assays were not statistically significant (Paired t-test; t_5_ = −1.35, P = 0.18) (Fig. 2a). Blue and green lights attracted more *D. suzukii* than red lights (ANOVA, F_2,117_ = 64.61, P < 0.0001; Tukey HSD, blue:green: P = 0.36, blue:red: P < 0.0001, green:red: P < 0.0001) (Fig. 2b).

### Preference among solid colors

**Figure 3:**
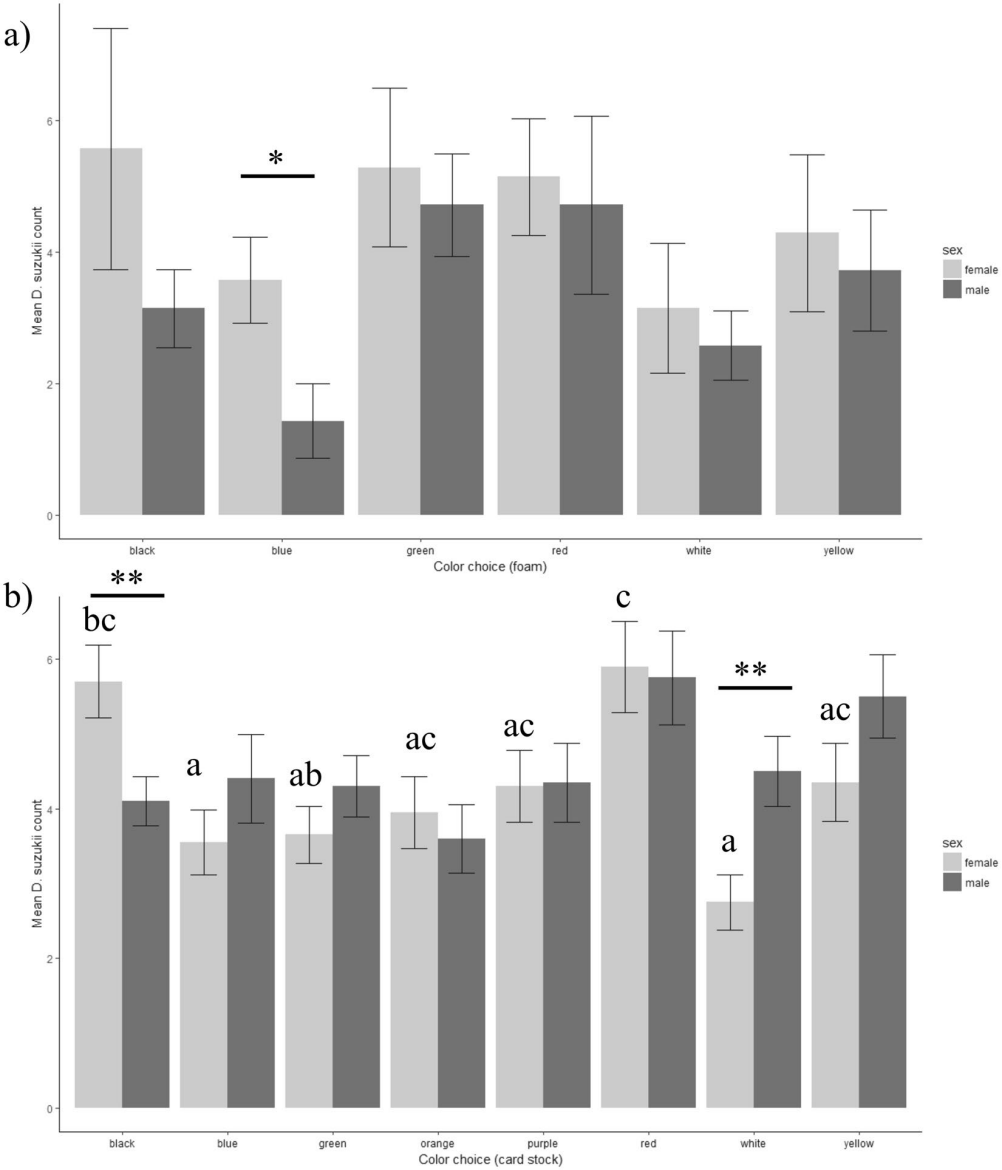
(**a**) Mean count (±SE) of *D. suzukii* attracted to foam board colors during six-color choice assays (1-way ANOVA, females: F_5,36_ = 0.70, P = 0.63; males: F_5,36_ = 2.31, P = 0.06). (**b**) Mean count (±SE) of *D. suzukii* attracted to card stock colors during eight-color choice assays (1-way ANOVA, females: F_7,152_ = 5.06, P < 0.0001; males: F_7,152_ = 2.01, P = 0.06). Significant difference between sexes for a given color are denoted by asterisks (paired t-test; *P ≤ 0.05, **P ≤ 0.001). Different letters above boxes represent statistically significant differences between color contrast discs for female flies and different letters below boxes represent significant differences for male flies (Tukey Post-Hoc, P ≤ 0.05).

Although higher numbers of *D. suzukii* were attracted to green, red, and black bands, differences observed among foam colors during choice assays were not significant (2-way ANOVA: color: F_5,72_ = 2.09, P = 0.08, sex: F_1,72_ = 3.56, P = 0.06, color:sex: F_5,72_ = 3.92, P = 0.85) (Fig. 3a). Mean responses to solid foam colors by female *D. suzukii* were higher than responses by males (paired t-test, t_5_ = 3.01, P = 0.03). Although numerical differences were observed between males and females for all colors, differences were significant only at blue (Welch’s 2-sample t-test, t_11.8_ = 2.48, P = 0.03). No correlations were observed between mean number of flies choosing a color (color choice) and percentage reflectance at any wavelength for foam colors (Spearman’s rank correlation, Females: P’s > 0.19; Males: P’s > 0.13).

However, in an expanded choice assay using cardstock colors, more female *D. suzukii* were found adhered to black and red, and more male *D. suzukii* were adhered to red and yellow (Fig. 3b). Preferences between red and black cardstock in female *D. suzukii* and among all card stock colors in male flies did not differ significantly (Fig. 3b). Differences in attraction between sexes were not significant overall (paired t-test, t_7_ = −0.80, P = 0.45; 2-way ANOVA: Color: F_7,304_ = 4.65, P < 0.0001; Sex: F_1,304_ = 1.44, P = 0.23; Color:Sex: F_7,304_ = 2.24, P = 0.03); however, significant differences were observed in responses to white and black cardstock (Fig. 3b). Differences in color preference were significant only within females, not within males (1-way ANOVA: Females: F_7,152_ = 5.06, P < 0.0001; Males: F_7,152_ = 2.01, P = 0.06) (Fig. 3b). Female color choice was inversely correlated with percentage reflectance at blue (470 nm; Spearman’s rank correlation; r_s_ = −0.39, P < 0.0001), cyan (525 nm; r_s_ = −0.32, P < 0.0001), and green (560 nm; r_s_ = −0.23, P < 0.005), but not at other wavelengths. No correlations were observed between male color choice and percentage reflectance (Spearman’s rank correlation, P’s > 0.11).

### Preference among color contrast discs

#### Color contrast assay 1

**Figure 4:**
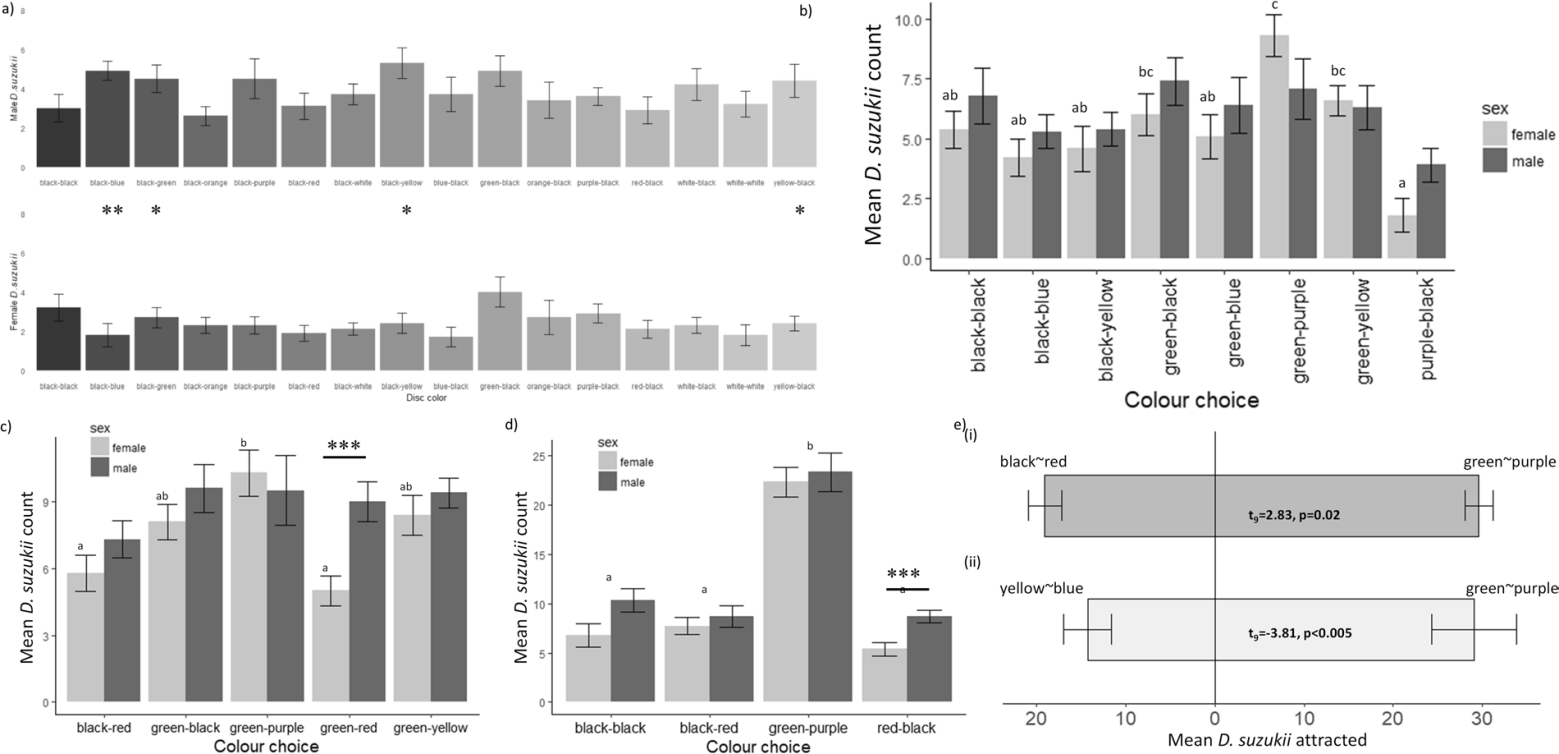
(**a**) Mean counts (±SE) of female and male *D. suzukii* attracted to colored discs in multi-choice trials (color contrast assay 1). (1-way ANOVA, females: F_15,154_ = 1.22, P = 0.26; males: F_15,154_ = 1.25, P = 0.24). The first color in each pair represents the outer ring color and the second represents the inner color ring (**b**) Mean count (±SE) of *D. suzukii* attracted to colored discs in multi-choice trials (color contrast assay 2) (1-way ANOVA, females: F_7,72_ = 6.81, P < 0.0001; males: F_7,72_ = 1.39, P = 0.22). (**c**) Mean count (±SE) of *D. suzukii* attracted to colored discs in multi-choice trials (color contrast assay 3) (1-way ANOVA, females: F_4,44_ = 6.07, P < 0.001; males: F_4,44_ = 0.82, P = 0.52). (**d**) Mean count ( ± SE) of *D. suzukii* attracted to colored discs in multi-choice trials (color contrast assay 4) (1-way ANOVA, females: F_3,36_ = 51.38, P < 0.0001; males: F_3,36_ = 28.94, P < 0.0001). Significant difference between sexes for a given color in figures a-d are denoted by asterisks (in figure a: paired-sample Wilcoxon test, P < 0.05 and in figures b-d: paired t-test, P < 0.05; *P ≤ 0.05, **P ≤ 0.01, ***P ≤ 0.001). Different letters above boxes represent statistically significant differences between color contrast discs for female flies and different letters below boxes represent significant differences for male flies (Tukey Post-Hoc, P ≤ 0.05). (**e**) Mean count ( ± SE) of *D. suzukii* attracted to colored discs in 2-choice trials. Results of black~red versus green~purple discs (color contrast assay 5) are shown in the top bar and results of yellow~blue versus green~purple discs (color contrast assay 6) are shown in the bottom bar. Results of paired t-tests are shown within the figure.

During the color contrasting-color assays with eight colors paired with color contrastblack, differences among color contrast discs within each sex were not statistically significant (2-way ANOVA: color: F_15,288_ = 1.49, P = 0.11; sex: F_1,288_ = 41.22, P < 0.0001; color:sex: F_15,288_ = 1.00, P = 0.46) (Fig. 4a). Responses to color contrast discs were significantly different between male and female *D. suzukii* (Paired t-test, t_159_ = −6.31, P < 0.0001) (Fig. 4a). Female *D. suzukii* were most attracted to discs with green as the outer color of the disc and black as the outer color of the disc; however, male *D. suzukii* were most attracted to discs with yellow or blue as the inner color and black as the outer color of the disc (Fig. 4a); however, differences were not significant (ANOVA, F_7,152_ = 1.77, P = 0.10). 82.6% of female flies and 89.3% of male flies captured were found on outer portions of the color contrast discs.

We conducted an ANOVA using the disc colors of the adjacent discs. There was no significant difference in attraction to any disc due to colors of adjacent discs for either sex (1-way ANOVA, Females: F_15,304_ = 1.48, P = 0.11; Males: F_15,304_ = 0.66, P = 0.82). No correlation was observed between percentage reflectance of disc outer colors (Spearman’s rank correlation, Females: P’s > 0.56; Males: P’s > 0.28), inner disc colors (Females: P’s > 0.08; Males: P’s > 0.44), or color contrast scores (Females: P’s > 0.35; Males: P’s > 0.46). n-numbers necessary to conduct more detailed analyses of where flies did not alight were deemed excessive.

#### Color contrast assay 2

Responses to color contrast-color discs were not significantly different between male and female *D. suzukii* (Paired t-test, t_79_ = −1.57, P = 0.12; 2-way ANOVA, color: F_7,144_ = 6.15, P < 0.0001; sex: F_1,144_ = 2.41, P = 0.12; color:sex: F_7,144_ = 1.13, P = 0.35) (Fig. 4b). Among color contrast-color discs comprised of the five most attractive colors in the previous assay (black, blue, green, purple, and yellow), discs of green~purple were most attractive to female *D. suzukii* (ANOVA, F_7,72_ = 6.81, P < 0.0001; Fig. 4b), although differences between green~purple and green~black and between green~purple and green~yellow were not significant. No significant differences in male *D. suzukii* preferences for color contrasting-color discs were observed (1-way ANOVA, F_7,72_ = 1.39, P = 0.22) (Fig. 4b). 85.1% of female flies and 88.5% of male flies captured were found on outer portions of the color contrast discs.

Discs with green outer rings attracted the most *D. suzukii* (1-way ANOVA, F_2,157_ = 15.55, P < 0.0001) (Fig. 4b). Differences in attraction between sexes were not significant (2-way ANOVA, outer color: F_2,154_ = 15.73, P < 0.0001; sex: F_1,154_ = 2.33, P = 0.13; color:sex: F_2,154_ = 1.23, P = 0.30) (Fig. 4b). Purple was the most attractive color of inner ring on color contrast discs for female *D. suzukii* (1-way ANOVA, F_3,76_ = 8.03, P = 0.0001) (Fig. 4b). No significant differences in preference for inner ring color were found for male *D. suzukii* (1-way ANOVA, F_3,76_ = 0.42, P = 0.74). Disc colors of adjacent discs were not associated with any significant differences in preferences for either sex *D. suzukii* (1-way ANOVA, Females: F_7,152_ = 0.30, P = 0.95; Males: F_7,152_ = 0.42, P = 0.89) (Fig. 4b). Attraction to discs in female *D. suzukii* was correlated to percentage reflectance values for blue (470 nm) to yellow (585 nm) for outer colors (Spearman’s rank correlation, P’s < 0.005), for yellow (585 nm) to deep red (700 nm) for inner colors (P’s < 0.02), and for orange (600 nm) to deep red (700 nm) for color contrast between outer and inner discs (P’s < 0.01). No correlations were observed for responses of male *D. suzukii* to percentage reflectance or color contrast scores (outer color: P’s > 0.21; inner color: P’s > 0.38, color contrast score: P’s > 0.19).

#### Color contrast assay 3

Responses to color contrast-discs were significantly different between male and female *D. suzukii* (Paired t-test, t_48_ = −2.37, P = 0.02; 2-way ANOVA, color: F_4,88_ = 4.26, P < 0.005; sex: F_1,88_ = 5.15, P = 0.03; color:sex: F_4,88_ = 1.52, P = 0.20) (Fig. 4c). Green~purple discs were most attractive to female *D. suzukii* (Fig. 4c; ANOVA, F_4,44_ = 6.07, P < 0.001). No significant differences in preference were observed for male *D. suzukii* (1-way ANOVA, F_4,44_ = 0.82, P = 0.52) (Fig. 4c). Discs with green outer rings were more attractive than discs with black outer rings, although not significantly so for male *D. suzukii* (1-way ANOVA, all flies: F_1,96_ = 7.16, P < 0.01;, Females: F_1,47_ = 4.09, P = 0.05; Males: F_1,47_ = 3.31, P = 0.08) (Fig. 4c). Disc colors of adjacent discs were not associated with any significant differences in preferences for either sex (1-way ANOVA, Females: F_4,93_ = 0.39, P = 0.82; Males: F_4,93_ = 0.90, P = 0.47) (Fig. 4c).

#### Color contrast assay 4

Responses to colored discs were different between sexes (paired t-test, t_39_ = −3.10, p < 0.005; 2-way ANOVA, color: F_3,72_ = 75.72, P < 0.0001; sex: F_1,72_ = 6.58, P = 0.01; color:sex: F_3,72_ = 0.66, P = 0.58) (Fig. 4d); therefore, results were calculated separately for each sex. Discs with a green outer ring and purple inner ring (green~purple) attracted higher numbers of both male and female *D. suzukii* than did black discs or discs combining red and black (1-way ANOVA, all flies: F_3,76_ = 71.44, P < 0.0001; females: F_3,36_ = 51.38, P < 0.0001;males: F_3,36_ = 28.94, P < 0.0001) (Fig. 4d). 87.2% of female flies and 88.6% of male flies captured were found on outer portions of the color contrast discs.

#### Color contrast assay 5

In two-choice assays between black~red discs and green~purple discs, responses were not different between sexes (Paired t-test, t_1_ = 0−68, p = 0.62); therefore, results for both sexes have been combined. Both male and female *D. suzukii* were attracted in higher numbers to green~purple discs than black~red discs (Fig. 4e (i)).

In two-choice assays between green~purple discs and yellow~blue discs, responses were not different between sexes (paired t-test, t_1_ = 0−83, p = 0.56); therefore, results for both sexes have been combined. Both male and female *D. suzukii* were attracted in higher numbers to green~purple discs than yellow~blue discs (Fig. 4e (ii)).

**Figure 5:**
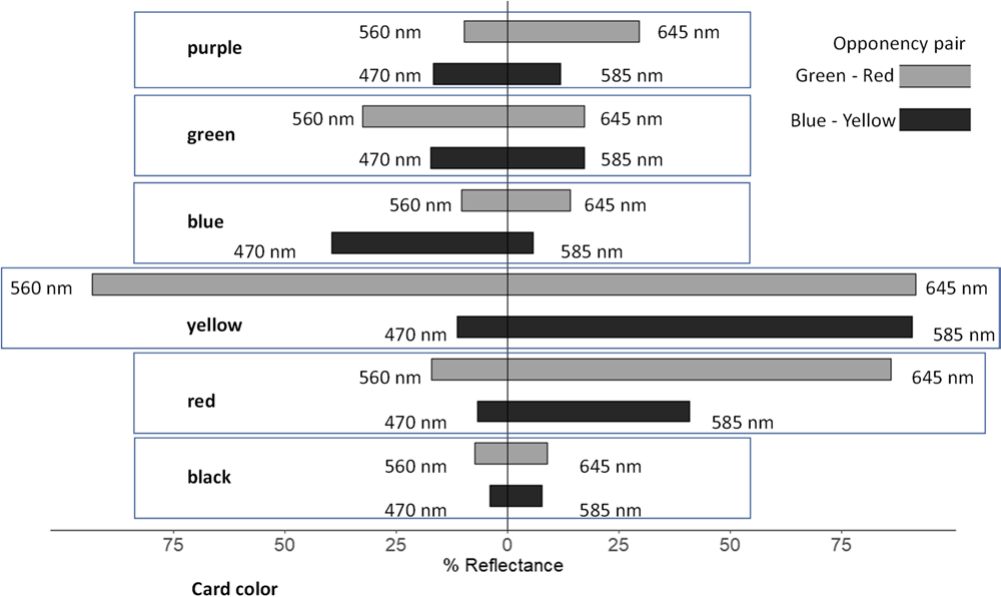
Comparison of the reflectance spectra for black, blue, green, yellow, purple, and red cardstock colors used in 2-choice assays of contrast color discs.

Analysis of the color spectra for colors used in choice assays shows that black cardstock had characteristically low reflectance at all wavelengths (Fig. 5). Comparison of reflectance spectra values at wavelengths thought to be important for color opponency revealed that red cardstock showed comparatively more reflectance at 585 nm and 645 nm wavelengths (yellow and red) than at 470 nm and 560 nm wavelengths (blue and green) (Figs 5 and S2). The center red portion of black~red discs reflects more light at all wavelengths than the outer black portion of the disc (Figs 5 and S2).

Yellow cardstock had high but comparatively equal reflectance values at 560 nm and 645 nm wavelengths (green and red), but higher reflectance at 585 nm (yellow) than at 470 nm (blue) wavelengths (Figs 5 and S2). Reflectance was proportionately equal between 560 nm (green) and 645 nm (red) for blue cardstock, but relatively higher at 470 nm (blue) than at 585 nm (yellow). The outer yellow portion of yellow~blue discs reflected more light at all wavelengths except blue compared to the inner blue portion of the discs (Figs 5 and S2).

Green cardstock reflected relatively more light at 560 nm (green) than at 645 nm (red) (Figs 5 and S2). Reflectance values were higher at 585 nm (yellow) than at 470 nm (blue). Purple cardstock reflected more light at 645 nm (red) than at 560 nm (green) and reflected more light at 470 nm (blue) than at 585 (yellow). Higher reflectance at 560 nm (green) of the outer green portion of green~purple discs color contrasted with the higher reflectance at 645 nm (red) of the purple portion of the disc. In addition, higher reflectance at 585 nm (yellow) in the green portion of the green~purple discs color contrasted with higher reflectance at 470 nm (blue) in the purple portion (Figs 5 and S2).

Similar relationships in color contrast were observed in green~yellow and green~black discs from color contrast assay 3. Specifically, the yellow portion of green~yellow discs reflected proportionately higher at 585 nm (yellow) and lower at 470 nm (blue) than did the green portion of the disc (Figs 5 and S2). Percentage reflectance was higher for the yellow portion of discs at both at 560 nm (green) and 645 nm (red) than for the green portion of the disc. Percentage reflectance was lower for the black portion of green~black discs than for the green portion of the disc (Figs 5 and S2).

## Discussion

The spectral sensitivity range of invertebrates differs from that of humans. This study reinforces the principle that research into the sensory discrimination and preferences of non-human species must use objective measures, and not measures that are biased by human perceptions or based on color appearance based on human perception. Spectral sensitivity experiments demonstrate that color discrimination by dipterans may be limited to just four broad color categories: ultraviolet, purple, blue, and green^34,49^. In general terms, Dipteran spectral sensitivity would limit color discrimination to wavelengths less than 600 nm^50^. Color recognition and preference have been noted in *Drosophila melanogaster* with strong color discrimination and sensitivity occurring between 406 nm and 505 nm^41,50–55^. Our preliminary studies of *D. suzukii* suggest that its visual range is comparable to that of *D. melanogaster* and set the stage for the behavioural experiments which form the bulk of this study. Further research is required to provide an in-depth analysis of *D. suzukii* optical physiology.

Previous studies on *D. suzukii* attraction to color have gauged behavioural responses to single colors in choice assays against a uniform white or black background^25,28,56^. However, we find that the physiological responses of *D. suzukii* to colored light (strong responses to blue and green, and a weak response to red) are consistent with previous findings that *Drosophila* species perceive red poorly relative to other colors^38,41,51,52,57^. We observed greater sensitivity in *D. suzukii* to light at the shorter wavelength range (blue-green) of the spectrum than at longer wavelengths (red). We found no significant difference in between attraction to reflected light from red and attraction to black, orange, purple, or yellow in single color choice assays. It has been suggested that *Drosophila* species perceive red as something akin to a dull green or yellow-green^58,59^. Thus, attraction of *D. suzukii* to red may be in response to contrast between bright and dark, iridescence, or ultraviolet reflectance rather than color and suggests that perception of red by *D. suzukii* is unlike human color perception^34,59^.

As in other studies, we found that *D. suzukii* were attracted to red, black, and yellow targets^25,26,29,56,60^. However, the attraction to single color targets was correlated with reflectance at short wavelengths (blue [470 nm] and green [560 nm]), rather than the overall color that humans perceive. We also found strong attraction to green targets that was comparable to responses to red targets. Given the lack of visual sensitivity and visual discrimination at longer wavelengths (red [645 nm]) by *Drosophila* species, the common practice of pairing red and black results may result in decreased attractiveness in traps deployed for *D. suzukii*. We found that when given a choice between contrast discs, that discs pairing red and black offered no improvement in attraction compared to any other color combination we tested and attracted fewer *D. suzukii* than discs containing green.

We found that color combinations pairing green as a background color against other colors within the optimal sensitivity range of *Drosophila* species resulted in higher attractiveness. This is consistent with naturally occurring conditions for host-finding, where potential host fruits of various colors would normally be near, typically, green foliage. More than 80% of flies found on color contrast discs were located on the outer (background) ring of color. This consistent with behaviour we have observed in field settings where flies were observed to land on adjacent leaves before moving to fruits.

The color opponency model suggests that opposing values between blue and yellow and between green and red are important to color discrimination. Thus, visual color contrast is emphasized by pairing a shorter and longer wavelength as a binary system within each type of visual receptor neuron, such that each neuron can signal in response to only one of the two opposing color stimuli, not both, and that excitatory stimulation from one wavelength might be inhibitory to signals for the opposing wavelength color^37,47,61,62^. The green~purple color pairing preferred by *D. suzukii* in our experiments exploits this color opposition. The outer green portion has higher reflectance values at green (560 nm) than at red (645 nm), while having lower reflectance at blue (470 nm) than at yellow (585 nm). In comparison, the inner purple portion has lower reflectance values at green than at red, while having higher reflectance at blue than at yellow (Fig. 5). Consequently, green~purple should appear as high color contrast and a strong visual cue for *D. suzukii*. Color discrimination could be further improved by refining color choice so that peak reflectance at blue vs. yellow opposes reflectance at green vs. red in each color. Color contrast could be further refined by ensuring the color opposition pattern of inner and outer portions of the color contrast discs are the reverse of each other.

This study has provided physiological and behavioral evidence of color preferences of *D. suzukii*, and the ability of this species to discriminate between selected ranges of colors. However, other elements of visual cues may influence detection, perception and orientation in insect species. Achromatic features of a visual cue, such as brightness/intensity or contrast may also be important in orientation and attraction. This study controlled for this using a select range of colors with comparable peak reflectance values. As well, detection and orientation to visual cues within the UV range has been shown in *D. melanogaster*^44^. Future work should investigate the roles of achromatic cues and expanded wavelengths of light for potential behavioral and physiological impacts on *D. suzukii* and other Drosophilids.

For both feeding and oviposition, *D. suzukii* must locate small ripening fruits and berries of various colors within a background of predominantly green foliage. We have previously demonstrated that *D. suzukii* use color contrast in color between ripening fruits and surrounding foliage to identify suitable host fruits^23^. While olfactory cues are the primary driver of host-finding behaviour in many *Drosophila* species and thought to be the primary driver in *D. suzukii*, we have presented evidence to suggest that color can play a significant role in host-finding and potentially other behaviours^20,63–66^. Differences in reflectance within opponent color pairs (green vs. red and blue vs. yellow) contributes to color discrimination in *D. suzukii* and these differences promote host-finding through color contrast between foreground (fruit) and background (foliage) colors.

## Methods

### *D. suzukii* colony

Adult *D. suzukii* flies used for all laboratory experiments were sourced from colonies maintained at Acadia University, Wolfville, NS since 2013. Initially, *D. suzukii* used to found colonies were reared from cultivated blueberries by D. Moreau at the Kentville Research and Development Centre (Agriculture and Agri-Food Canada, Kentville, NS). Colonies were maintained in 250 ml flasks (Genesee Scientific, San Diego, CA) containing 50 mL of Formula 4–24 Instant *Drosophila* medium (Merlan Scientific Ltd, Mississauga, ON, Canada) mixed with 50 mL of dH2O. Sexually-mature mated *D. suzukii*, approximately two weeks of age, were removed from colony vials and starved for 2 h prior to start of each assay.

### Sensitivity to color (Electroretinography)

Color sensitivity differs among insect orders and even among many species; however, the color vision system in flies (Diptera) is believed to be relatively well conserved^38^. The visual system of *D. melanogaster* has been extensively studied^34–41,43,50,51,53,67–73^. We conducted preliminary studies of *D. suzukii* visual physiology to confirm its visual ranges were consistent with *D. melanogaster* and inform the behavioural experiments that follow. We tested sensitivity of female and male *D. suzukii* to blue, green, and red light-emitting diode (LED) lights and a full spectrum white LED light using a Bluetooth-enabled Programmable BeeWi 9 W SmartLite LED Colour Bulb and SmartPad app (VOXLAND, Marseilles, France). Analysis of light spectra for each LED light color were conducted with advice and assistance of Dr. Michael Robertson (Department of Physics, Acadia University), who specializes in optics and optical properties. Spectra for each color light were measured using an Ocean Optics USB4000 Spectrometer (corrected linearity > 99%) and SpectraSuite Spectrometer Operating Software (Ocean Optics, Inc., Dunedin FLA) (Fig. S1a). We note that the blue LED light which the authors perceived as blue had a maximum peak value in the UV range, but the peak range extended from ultraviolet into blue wavelengths (Fig. S1a). A similar pattern was observed for the green LED light, with a maximum peak value in at a wavelength consistent with blue-green (cyan) and a peak range extending from ultraviolet to yellow wavelengths (Fig. S1a). Nine replicates of blue wavelength spectra and ten replicates of green and red wavelength spectra were measured to ensure consistency of light color (One-Way ANOVA; Blue: F_8,9387_ = 0.40, P = 0.92; Green: F_9,10430_ = 0.21, P = 0.99; Red: F_9,10430_ = 0.77, P = 0.64). Lights were set at maximum brightness of 756 lumens for all assays. Light intensity was comparable across white and colored lights (Fig. S1a). Intermediate colors pink, turquoise, and yellow could also be emitted by BeeWi lights; however, these colors were achieved using a combination of blue, green, and red LED lights, not by emitting intermediate wavelengths, and so were not used for testing.

Changes in sensory receptor neuron activity were measured with electroretinograms^74^ using an IDAC-2 signal connection controller and GC-EAD 2014 × 1.2.5 software (Syntech Data Acquisition for Gas Chromatography with EAD, Syntech Equipment and Research, Kirschzarten Germany). Individual *D. suzukii* were mounted in 200 µl pipette tips, allowing only the head to emerge (Fig. S1b). All overhead laboratory lighting was extinguished once set-up was complete and not switched on until after the assay was complete. Each *D. suzukii* preparation was acclimatized for 10 minutes prior to start of electroretinogram assays.

The light source was enclosed within a cardboard box and light was directed at the fly’s eye through a 12 mm × 12 mm hole covered by a flap of black foam-board. Light colors were changed with the box closed and flies were exposed to light colors in random order at one-minute intervals. Each fly was exposed to white light at the beginning, middle, and end of the trial as a positive control. Blue, green, and red lights were presented in random order twice during each trial. Eleven replicates were completed for each sex of fly, using a naïve fly for each replicate.

### Preference among LED light colors

*Drosophila suzukii* preference among blue, green, and red light was assessed through two-choice assays using the same LED color bulbs as in the electroretinograms. Light intensity was consistent among light colors (Fig. S1a). Mean intensity levels at spectral peaks are white: 58295 counts/ms at 449.46 nm, blue: 51471.3 counts/ms at 462.67 nm, green: 54904.9 counts/ms at 513.78 nm, and red: 51981.9 counts/ms at 629.47 nm. Arenas were constructed of 3-inch diameter (7.6 cm) black ABS (Acrylonitrile butadiene styrene) pipe fittings and cleanout T-fitting, using a modified set-up based on Diclaro *et al*.^75^ (Fig. S1c). Clear plastic sandwich bags coated with TangleTrap Sticky Coating (The TangleFoot Company, Grand Rapids, MI) were placed over plastic drinking cups covered with black duct tape that were fitted into 3-4-inch (7.6–10.2 cm) diameter ABS adapters at either end of the arena. Colored light was directed perpendicularly into the arena via a small 2 × 2 cm clear openings on the side of each cup to prevent flooding the arena with light and prevent blinding the insects. The position of each light color was alternated relative to the other from one trial to the next to mediate positional effects. Phototaxis combined with differential sensitivity to the wavelength ranges of each color light was expected to influence attraction choices. Male and female *D. suzukii* were tested separately. Twenty-five mature *D. suzukii* were inserted through the port located at the center of the arena (Fig. S1c, position A). Each paired color choice was replicated 10 times for each sex. After 24 h, *D. suzukii* adhering to the TangleTrap at each end of the arena were counted.

### Preference among solid colors

Two cylindrical arenas were constructed using vertical strips of colored foam arranged around the circumference of an 11.8 L plastic container (circumference of 74 cm at top & 67 cm at bottom and height of 29.5 cm). Two strips each of black, blue, green, yellow, red, and white were repeated twice in each arena. Color order was arranged to ensure that adjacent colors were different in each arena (Fig. S2a, arenas 1 and 2). The colored foam surfaces were covered with clear cellophane tape and brushed with a 1.5 mm coating of TangleTrap Sticky Coating per package directions. No change in color reflectance was observed following application of sticky coating^23^. As *D. suzukii* alighted on a coated surface, they adhered to the colored strip. Male and female *D. suzukii* were tested separately. Fifty mature *D. suzukii* were placed at the center of the arena. The top of the container was covered with cellophane. The container lid was cut open, leaving only the outer rim, which was used to secure the cellophane. Arenas were placed inside a box to reduce ambient light. A clear plexiglass panel placed over the arena supported a full spectrum light source (VX Series High CRI LED 14 W Bulb, Yuji International, Beijing, China) that approximated daylight conditions (([5600 K daylight spectrum, CRI typical 97/100, TLCI typical 99/100 {https://www.yujiintl.com/high-cri-led-lighting.html}] VX Series High CRI LED 14 W Bulb, Yuji International, Beijing, China) and that illuminated the entire arena area. Natural field light conditions differ dependent upon time of day, time of year, geographic location, and other abiotic and biotic environmental conditions. It would not be feasible to replicate all possible natural light conditions. While CRI and TLCI scores are appearance measures for lighting based on human perception, we chose the VX Series High CRI LED bulb for its relatively consistent intensity values across the spectrum consistent with industry standard “natural light” values (380 nm to 750 nm) stated in the product specifications provided by the manufacturer (https://www.yujiintl.com/high-tlci-led7318.html?testrp). After 24 h, flies adhered to each of the colored strips were counted. Seven replicates of six-color trials were conducted for both male and female *D. suzukii*, alternating arena used for each trial.

Color options were limited using foam substrates, so we elected to conduct all subsequent assays using cardstock with a wider range of color choices. Multi-choice assays were also conducted in two arenas using eight colors of card stock in the same type of arenas (Fig. S2a, arenas 3 and 4). Two strips each of black, purple, blue, green, yellow, orange, red, and white were repeated twice in each arena. Ten replicates of eight-color trials in each arena were conducted for both male and female *D. suzukii*.

Reflectance spectra for each color of foam and cardstock were measured with Alta II reflectance spectrometer (Vernier Software & Technology, Beaverton, OR USA) to quantify colors used (Fig. S3). We used spectrometer measures of reflectance at 7 wavelengths covering a range of 470 nm to 700 nm.

### Preference among color contrast discs

Color contrasting color discs, based on those used by Kirkpatrick *et al*.^28^, were constructed of pairs of card stock discs 5 cm in diameter overlaid with cardstock discs of 2.5 cm diameter (same front and back of disc). Each disc was covered with clear cellophane tape and coated with TangleTrap Sticky Coating. Discs were suspended from the top of a 30 × 30 × 30 cm plastic and mesh insect cage (BugDorm, MegaView Science Co. Ltd., Talchung, Taiwan). Discs were arranged in random order, equidistant from each other, and at a radius of 12.5 cm from the center of the cage. We recorded the order of the discs around the arena. Ambient light and external visual distractions were excluded from each arena with white cardboard trifold display boards. We have approximated field light conditions by illuminating arenas with a full spectrum light source ([5600 K daylight spectrum, CRI typical 97, TLCI typical 99] VX Series High CRI LED 14 W Bulb, Yuji International, Beijing, China). Female and male *D. suzukii* were tested separately. One hundred mature *D. suzukii* were released into the center of each arena. After 24 h, *D. suzukii* adhering to each disc were counted. Results of each set of assays were used to inform and refine the color choices for the next iteration.

#### Color contrast disc assay 1

Each of the eight colors used in solid color multi-choice assays were used in color contrast with black (Fig. S2b). Color contrast pairs are hereafter denoted as “outer color ~ inner color” for clarity. Colors were paired with black as either foreground (inner portion) or background (outer portion of disc) to identify possible effects of color contrast inversions.

#### Color contrast disc assay 2

Color contrasting color assays paired combinations of black, blue, green, purple, and yellow based on mean fly counts in the previous assay (Fig. S2c). Fruits in a field setting would normally be observed against a background of foliage, which is typically green. Therefore, we tested green as a background color rather than a foreground color. The most commonly used monitoring traps for *D. suzukii* use a combination of red against black, therefore, we also tested black as a background color.

#### Color contrast disc assay 3

Based on results of color contrast disc assay 2, assays were conducted with four color contrasting color discs of green background with black, purple, red, and yellow and a fifth disc of black~ red representing the colors used most frequently for monitoring traps (Fig. S2d).

#### Color contrast disc assay 4

To simplify subsequent assays, we have focused on the color combination with the highest mean attraction (green-purple), although differences in attraction compared to green-yellow and green-black were not significant. To account for potential differences in attraction due to color contrast inversions between black and red, discs of green~ purple were tested against black discs and discs that combined red and black (Fig. S2e).

#### Color contrast disc assay 5

To ensure differences in counts were not attributable to differences in availability between color options, green~ purple discs were paired against black~ red discs in two-choice assays (Fig. S2f). Because results of choice assays to this point revealed the importance of blue and yellow reflectance on behaviour, green~ purple discs were also paired against yellow~blue discs (Fig. S2g).

Ten replicates were completed for each sex and each multi-choice assay (assays 1-4) and five replicates for each sex for two-choice assays (assay 5).

### Statistical analysis

A color contrast score was calculated based on the Weber color contrast for percentage reflectance at each wavelength for each color-color contrast disc (Table S1)^76^.

$$Color\,contrast=({I}_{i}-{I}_{o})/{I}_{o}$$

I_i_ is reflectance (%) of inner ring. I_o_ is reflectance (%) of outer ring.

Statistical tests used for data analyses are described in the results for each assay. Responses of male and female flies were analyzed separately due to potential sex-specific physiology and behaviour. Analyses of choice assays were adapted from Kirkpatrick *et al*.^28^. All statistical analyses were performed in R version 3.4.3 (R Core Team 2017 [RStudio Version 1.1.419 - © 2009–2018 RStudio, Inc.]).

### Ethical approval

This article does not contain any studies with human participants or animals 443 performed by any of the authors.

## Supplementary information

Supplementary information

## Data Availability

Data has been stored as open access at: 10.5061/dryad.vhhmgqnpn.
