# Supplementary material for: Color preference of the spotted wing Drosophila, Drosophila suzukii
Source: Sci Rep. 2019 Nov 5;9:16051. doi: 10.1038/s41598-019-52425-w (PMC6831584; doi:10.1038/s41598-019-52425-w)
Supplement: Supplementary file 1 — Supplementary information [file 41598_2019_52425_MOESM1_ESM.pdf]

Catherine M. Little
Acadia University, 33 Westwood Ave, Wolfville,
Nova Scotia, Canada B4P2R6
Phone: 1.902.681.355
Email: cate.little@acadiau.ca or clittle@mun.ca

Color preference of the spotted wing Drosophila, *Drosophila suzukii*

Catherine M. Little[a,b], A. Rebecca Rizzato[a], Lise Charbonneau[a], Tom. Chapman[b], and N. Kirk Hillier[a]

Affiliations

[a] Department of Biology, Acadia University, Wolfville NS Canada B4P2R6

[b] Department of Biology, Memorial University of Newfoundland and Labrador, St. John's NL Canada A1C5S7

ORCID

Catherine M. Little #0000-0002-7389-9948

N. Kirk Hillier #0000-0002-0911-0332

Supplementary information

Table S1. Percentage reflectance of (a) foam and (b) cardstock colors and (c) contrast scores for color contrast discs made from cardstock and used in color contrast assays.

a) Foam colors

disc	blue (470 nm)	cyan (525 nm)	green (560 nm)	yellow (585 nm)	orange (600 nm)	red (645 nm)	deep red (700 nm)
black	1.66	2.54	3.35	2.84	2.67	3.93	2.47
blue	34.33	7.96	7.81	6.39	6.98	16.81	16.62
green	19.65	75.84	75.45	51.87	41.16	42.91	51.03
red	4.75	4.53	15.29	37.18	58.37	84.39	85.80
white	75.61	90.11	100.56	98.52	97.67	94.99	92.16
yellow	16.56	94.09	107.25	103.26	101.74	97.14	93.81

b) Cardstock colors

disc	blue (470 nm)	cyan (525 nm)	green (560 nm)	yellow (585 nm)	orange (600 nm)	red (645 nm)	deep red (700 nm)
black	3.97	5.42	7.25	7.82	7.44	8.94	7.31
blue	39.51	14.93	10.16	5.92	5.47	14.18	14.97
green	17.22	40.91	32.48	17.41	11.98	17.25	17.56
orange	11.59	24.21	37.95	68.8	81.28	85.94	84.62
purple	16.56	6.19	9.71	11.96	13.95	29.56	36.42
red	6.62	6.52	17.08	40.85	64.3	86.17	85.09
white	80.46	85.57	92.63	89.76	90.58	92.61	90.28
yellow	11.26	83.8	93.19	90.94	90.58	91.66	88.16

c) Contrast scores using cardstock colors

disc	blue (470 nm)	cyan (525 nm)	green (560 nm)	yellow (585 nm)	orange (600 nm)	red (645 nm)	deep red (700 nm)
black-black	0.00	0.00	0.00	0.00	0.00	0.00	0.00
black-blue	8.95	1.75	0.40	-0.24	-0.26	0.59	1.05
black-green	3.34	6.55	3.48	1.23	0.61	0.93	1.40
black-orange	1.92	3.47	4.23	7.80	9.92	8.61	10.58
black-purple	3.17	0.14	0.34	0.53	0.88	2.31	3.98
black-red	0.67	0.20	1.36	4.22	7.64	8.64	10.64
black-white	19.27	14.79	11.78	10.48	11.17	9.36	11.35
black-yellow	1.84	14.46	11.85	10.63	11.17	9.25	11.06
blue-black	-0.90	-0.64	-0.29	0.32	0.36	-0.37	-0.51
green-black	-0.77	-0.87	-0.78	-0.55	-0.38	-0.48	-0.58
green-purple	-0.04	-0.85	-0.70	-0.31	0.16	0.71	1.07
green-red	-0.62	-0.84	-0.47	1.35	4.37	4.00	3.85
green-yellow	-0.35	1.05	1.87	4.22	6.56	4.31	4.02
orange-black	-0.66	-0.78	-0.81	-0.89	-0.91	-0.90	-0.91
purple-black	-0.76	-0.12	-0.25	-0.35	-0.47	-0.70	-0.80
red-black	-0.40	-0.17	-0.58	-0.81	-0.88	-0.90	-0.91
white-black	-0.95	-0.94	-0.92	-0.91	-0.92	-0.90	-0.92
white-white	0.00	0.00	0.00	0.00	0.00	0.00	0.00
yellow-black	-0.65	-0.94	-0.92	-0.91	-0.92	-0.90	-0.92

Supplementary Figures.

Fig. S1. a) Mean spectra were calculated from measured wavelength emissions of blue, green, red, and white lights emitted from BeeWi SmartLite® bulb. b) A ground electrode comprised of a glass electrode containing a tungsten wire filament and insect saline was inserted at the base of the *Drosophila*'s head. A sharpened tungsten wire probe was inserted into the Drosophila's right eye at a 90° angle to act as the recording electrode. c) Arena set up for 2-choice light attraction assay (Diagram not to scale). *D. suzukii* were released into center of arena (position A). Light was directed perpendicularly into the arena through clear ports at either end of the arena. *Drosophila suzukii* attracted to the lights were trapped on Tangle-Trap coated clear plastic (black lines).

Fig. S2. a) Setup of choice assays used to determine color preferences. *Drosophila suzukii* were released into arenas with six different colors of foam board (arenas 1 & 2) and with eight different colors of card stock (arena 3 & 4). Two-color discs were hung in random order equidistant from the center of a 30 x 30 x 30 cm plastic and mesh cage. Discs in all choice assays were 5 cm in diameter with 2.5 cm diameter centers. b) Each of the eight colors of card-stock were used in combination with black for color contrast. c) Two-color discs comprised of black, green, blue, purple, and yellow were used for a second round of color contrasting color choice assays. d) A third round of multi-choice color contrast assays consisted of four green discs with centers of black, purple, red, and yellow and one black disc with a red center. e) Green discs with purple centers were tested against discs of black and red, the most commonly used colors for *D. suzukii* traps, and against black discs. Two-choice assays paired f) green~purple discs against black~red discs and g) green~purple discs against yellow~blue discs.

Fig. S3. Reflectance spectra for each color used in choice assays were measured. a) Six colors of foam board were used in multi-choice assays. b) Eight colors of card stock were used in multi-choice and 2-choice assays.

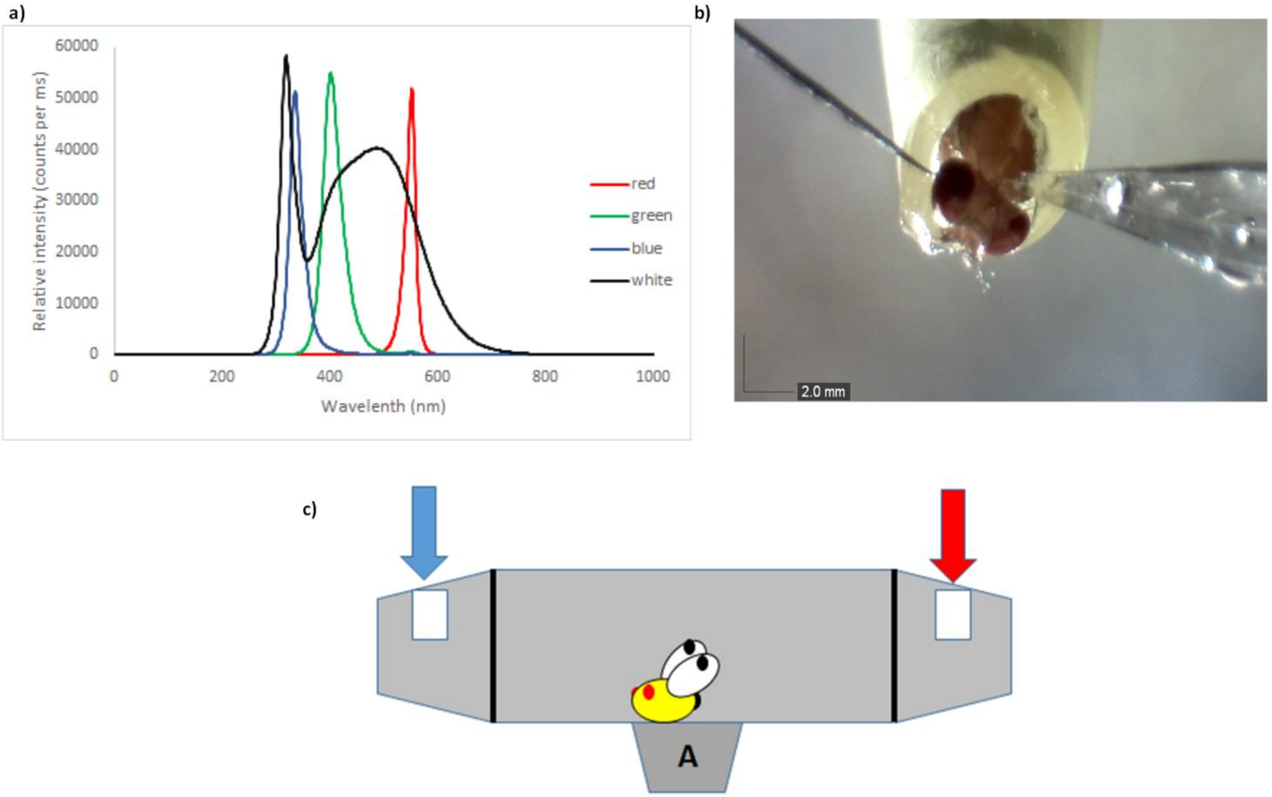
a)
60000
50000
40000
30000
20000
10000
0
Relative intensity (counts per ms)
0
200
400
600
800
1000
Wavelenth (nm)
red
green
blue
white
b)
2.0 mm
c)
A

Figure S1

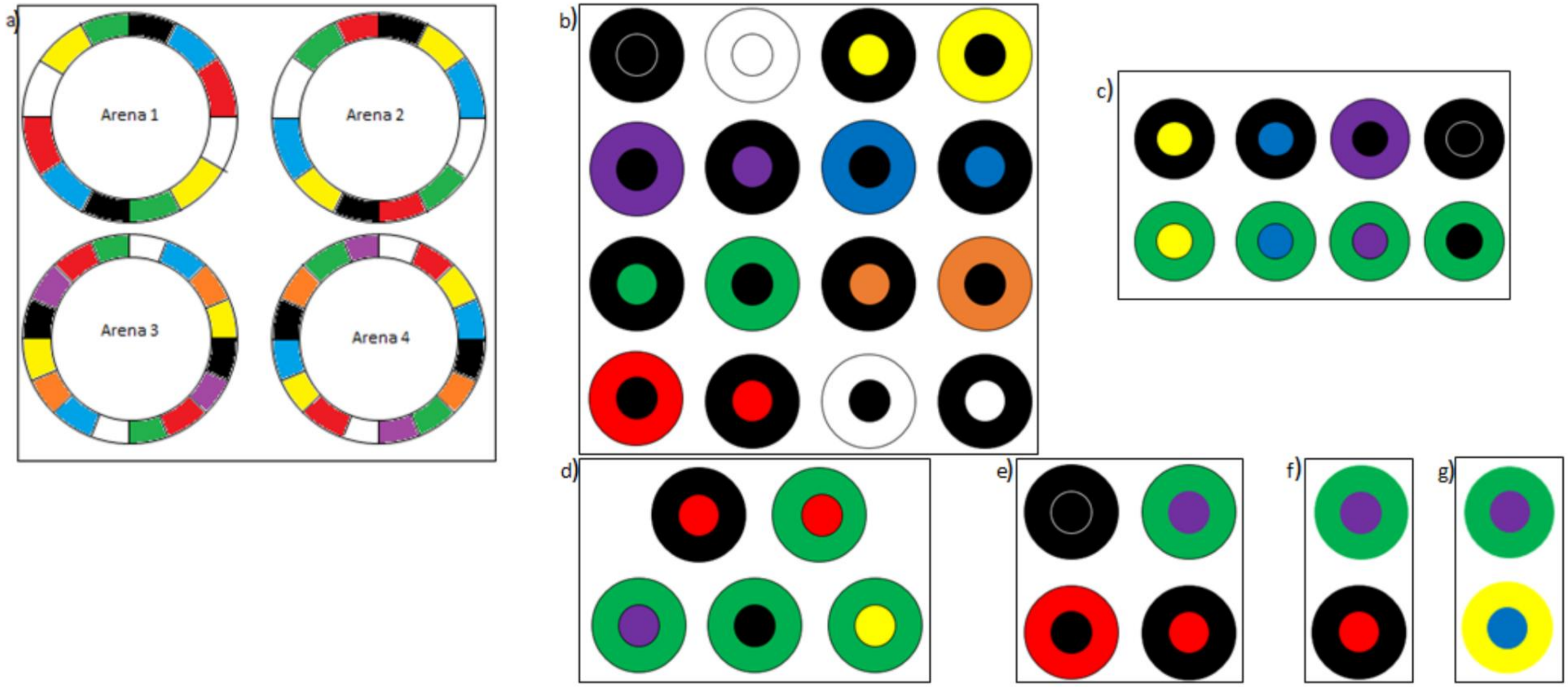

Figure S2

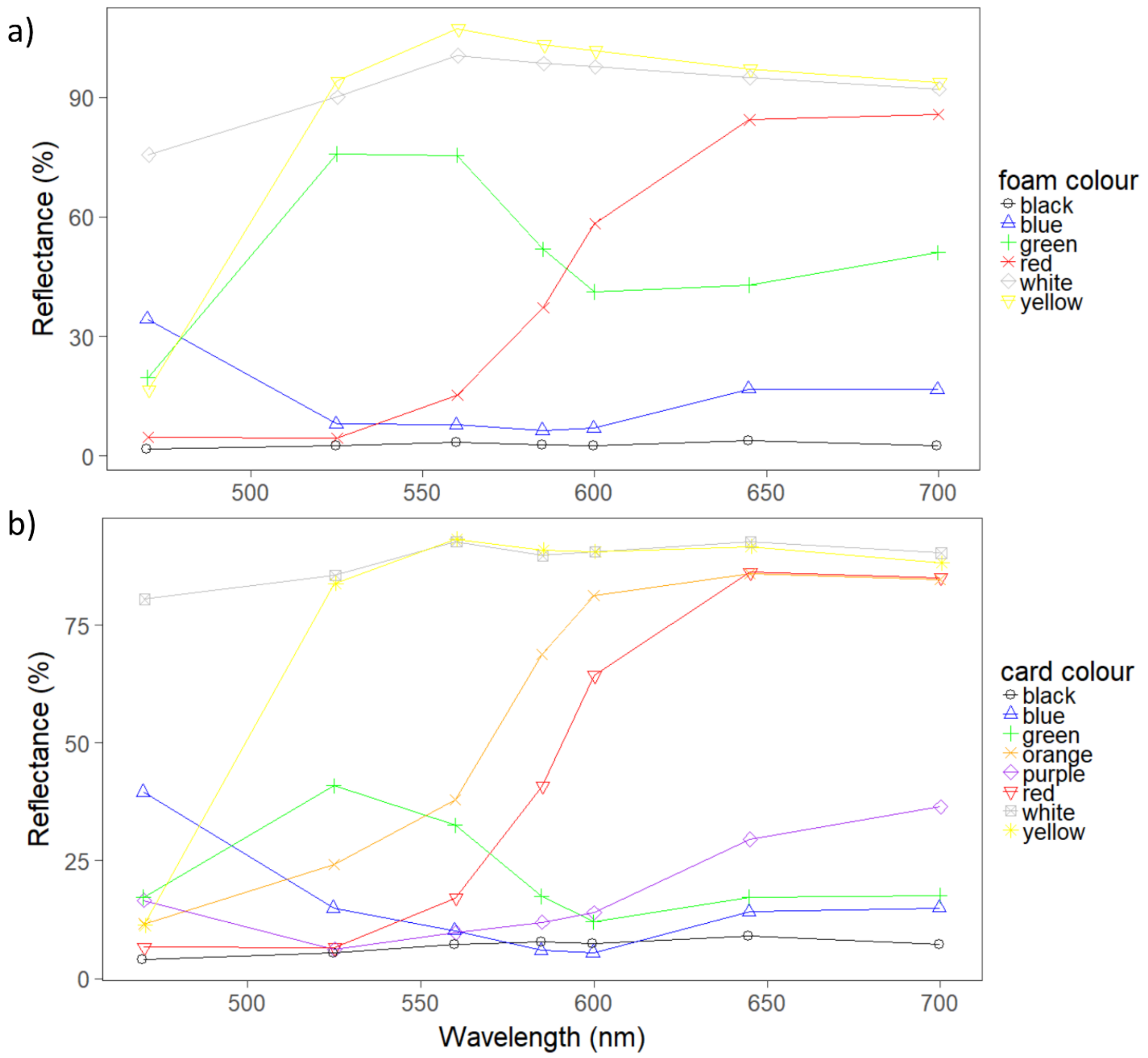
a)
Reflectance (%)
90
60
30
0
500
550
600
650
700
foam colour
black
blue
green
red
white
yellow
b)
Reflectance (%)
75
50
25
0
500
550
600
650
700
Wavelength (nm)
card colour
black
blue
green
orange
purple
red
white
yellow

Figure S3
